# Assessing Health Impacts of Conventional Centralized and Emerging Resource Recovery-Oriented Decentralized Water Systems

**DOI:** 10.3390/ijerph17030973

**Published:** 2020-02-04

**Authors:** Xiaobo Xue Romeiko

**Affiliations:** Department of Environmental Health Sciences, School of Public Health, University at Albany, State University of New York, Albany, NY 12222, USA; xxue@albany.edu

**Keywords:** health impacts, water and wastewater systems, life cycle assessment, microbial risk assessment, decentralization, resource recovery

## Abstract

Energy shortage and climate change call for sustainable water and wastewater infrastructure capable of simultaneously recovering energy, mitigating greenhouse gas emissions, and protecting public health. Although energy and greenhouse gas emissions of water and wastewater infrastructure are extensively studied, the human health impacts of innovative infrastructure designed under the principles of decentralization and resource recovery are not fully understood. In order to fill this knowledge gap, this study assesses and compares the health impacts of three representative systems by integrating life cycle and microbial risk assessment approaches. This study found that the decentralized system options, such as on-site septic tank and composting or urine diverting toilets, presented much lower life cycle cancer and noncancer impacts than the centralized system. The microbial risks of decentralized systems options were also lower than those of the centralized system. Moreover, life cycle cancer and noncancer impacts contributed to approximately 95% of total health impacts, while microbial risks were associated with the remaining 5%. Additionally, the variability and sensitivity assessment indicated that reducing energy use of wastewater treatment and water distribution is effective in mitigating total health damages of the centralized system, while reducing energy use of water treatment is effective in mitigating total health damages of the decentralized systems.

## 1. Introduction

Water and wastewater infrastructure is necessary and critical for providing the basic human needs of water and sanitation services. While centralized water and wastewater infrastructure has been successfully applied over many decades to provide adequate water and sanitation services in industrialized urban areas, centralized water and wastewater infrastructure is energy intensive and ranks as a significant contributor to greenhouse gas emissions (GHGs) [1]. To conserve energy use and combat climate change, alternative infrastructure design, under the principles of decentralized and resource recovery, are emerging in industrialized urban areas [2]. Though they are being implemented globally, the health impacts of decentralized and resource recovery-based infrastructure are not fully understood [3,4]. Sustainable development calls for simultaneously conserving energy, protecting environmental integrity, and promoting public health. In order to support sustainable development, it is critical to assess the health risks of the emerging decentralized and resource recovery-based infrastructure.

The majority of previous studies investigated health risks of water and sanitation services with either a life cycle assessment (LCA) or quantitative microbial risk assessment (QMRA) approach. LCA is a systematic evaluation approach, which is capable of quantifying environmental health impacts of chemical releases from a process, product, or service [5]. LCA includes chemical health risks caused by both treatment technologies and their supply chain processes, such as the production of the required energy and coagulant. The majority of the existing LCA studies focused on energy and water use, greenhouse gas emissions, and nutrient pollution [2,3,6]. A few recent LCA studies have begun to include human health impacts for urban water services. For example, Xue et al. investigated the life cycle cancer and noncancer impacts of water and wastewater treatment in Cincinnati [1,7,8]. Jeong et al. studied the life cycle cancer and noncancer impacts of water and wastewater treatment in Atlanta [9]. Lane et al. looked at the human toxicity of conventional centralized and emerging wastewater reuse systems [10]. While these studies are valuable, they primarily focused on life cycle health risks of centralized and semi-centralized systems. Meanwhile, a QMRA approach was applied to evaluate the pathogen risks associated with water and wastewater systems [3]. The existing literature assessed the pathogen risks of semi-centralized wastewater recovery for non-potable use, and decentralized wastewater treatment design or nutrient recovery [11]. Although these studies are valuable and insightful, these studies did not integrate LCA and QMRA approaches for holistically assessing health risks of water systems.

To the best of our knowledge, only four studies exist which couple LCA and QMRA for quantifying health risks of water systems [12,13,14,15]. Harder et al. assessed the health risks of sewage sludge management [13,14]. Kobayashi et al. quantified the health risks of a large-scale water recycling project in Sydney [15]. Anastasopoulou et al. investigated the health risks of self-sustained sanitation systems in South Africa [12]. Overall, the studies which couple LCA and QMRA for holistically assessing the human health impacts of water and sanitation systems are sparse, and no prior studies have applied an integrative LCA and QMRA approach to compare health risks of conventional centralized and decentralized resource recovery-based sanitation systems.

The goal of this study was to provide a comparative health risk assessment of three representative systems by integrating LCA and QMRA approaches. These three representative systems consisted of a conventional once-through centralized system and two emerging decentralized systems which are capable of conserving water and recycling nutrients. To the author’s best knowledge, this is the first study that compared the life cycle and microbial health impacts of centralized and decentralized water systems.

## 2. Method

### 2.1. Description of Evaluated Water and Wastewater Systems

The centralized wastewater system is defined in this study as a system that transports household wastewater to a centralized wastewater treatment plant that is far away from the serving households. The decentralized system is an on-site wastewater system that is not connected to a centralized wastewater treatment plant. The key difference between centralized and decentralized systems is the conveyance system for wastewater collection and transport. This study utilizes the centralized and decentralized wastewater system options in Falmouth, Massachusetts, in the United States (US) as case studies.

**Figure 1:**
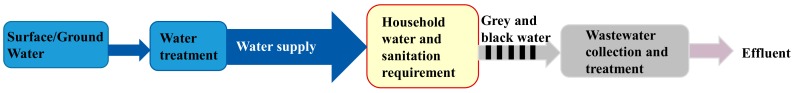
System description of three water system designs including business-as-usual (BAU), Composting Toilet-Septic Tank (CT-SS), and Urine Diverting Toilet-Septic Tank (UD-SS).

The Falmouth community, with a population of approximately 31,101 as of 2017, resides in the southeast tip of Cape Cod, Massachusetts State [16]. Falmouth consumes approximately 4.6 million gallons of water per day, approximately 60% of which is extracted from surface sources [17]. The Falmouth community is facing a eutrophication problem, primarily caused by the excessive nutrient releases from the septic tank systems predominately used for wastewater treatment. This study evaluated three community water and wastewater service options to replace current septic tank systems (Figure 1). All three assessed options were proposed by community stakeholders and have been demonstrated in other regions [18,19,20,21,22,23,24,25]. The first system, referred to as the business-as-usual (BAU), consisted of the existing centralized water service and a conventional once-through wastewater service system. The conventional once-through wastewater service system utilized an activated sludge treatment process and chlorine disinfection prior to discharge. The remaining two alternatives also maintained the centralized water system, but replaced the centralized wastewater system with decentralized and resource recovery systems. The first alternative system collected household urine and feces with dry composting toilets, and treated greywater with the existing septic system (CT-SS). Greywater included wastewater from sinks, showers, and washing machines within households. The second alternative system included urine-diverting toilets for capturing nutrients in urine, and the existing septic systems for treating grey and blackwater (UD-SS). Blackwater is toilet flushing water, including feces. The household water uses were provided by the existing centralized water treatment plant for the first two alternative systems. These two alternative systems maintained the existing centralized water supply systems and household septic tank systems, while enabling nutrient recovery from compost (urine and feces) or urine, respectively [26,27,28]. It is also worth noting that the two alternative systems are currently implemented at a pilot scale in Cape Cod. While BAU represents the centralized system, CT-SS and UD-SS represent the decentralized system.

### 2.2. Life Cycle Health Impact Assessment

As described by the International Organization for Standardization (ISO), LCA is a systematic evaluation approach consisting of four phases: goal and scope definition, inventory analysis, impact assessment, and interpretation [5]. The first step describes the assessed processes and identifies the system boundaries. The following step is to quantify energy, material usage, and environmental releases within the defined system boundaries. The third step is assessing the potential ecological effects of energy and material usage, and the environmental releases identified in the previous step. The last step aims to evaluate the results of the inventory analysis and impact assessment to understand data uncertainty and sensitivity, and to suggest the preferred scenario with the most preferred environmental performances. Each phase of LCA is described below.

#### 2.2.1. Goal and Scope

The goal of this study was to estimate life cycle health impacts of three representative systems, including BAU, CT-SS, and UD-SS. The scope of this study included both life cycle health impacts from each treatment process and their supply chain activities. The direct health impacts caused by environmental releases from the treatment processes during construction and operational stages were included. The indirect health impacts associated with the supply chain activities beyond the physical boundary of treatment processes, such as production of chemicals and electricity used in the treatment processes, were considered as well. To ensure an adequate comparison among resource recovery-based technologies and the conventional once-through system, the system scope of this study included both water and sanitation services. The water and wastewater services evaluated in this study began with water extraction and ended with wastewater discharge/reuse. The health impacts during the end-of-life handling of system components were excluded, due to lack of datasets.

The functional unit was Falmouth town’s annual water and wastewater service demand. Aligned with previous studies, this service-oriented functional unit reflected both water and wastewater treatment requirements, and aided in a fair comparison among the three systems [1,2,29]. Additionally, the avoided health impacts associated with the recovered energy and nutrients were estimated. For example, the equivalent amounts of fertilizer for compost from CT-SS and urine from UD-SS were estimated. The avoided life cycle health impacts were calculated based on the equivalent amount of the synthetic fertilizer and the life cycle health impacts of the synthetic fertilizer.

#### 2.2.2. Life Cycle Inventory

The existing water treatment plant in Falmouth utilizes the conventional processes, including alum coagulation, sedimentation, filtration with pH adjustment, and chlorine disinfection. The chemical and electricity demand of a water treatment plant and electricity use of water distribution were provided by local utilities [30]. The distributions of chemical and energy requirement are summarized in Table 1. Drinking water loss via the distribution system was considered to vary from 8% to 15%, in accordance with the national averages [31,32]. The wastewater treatment plant for BAU was considered to include sedimentation, secondary activated sludge treatment, tertiary nutrient removal, and chlorine disinfection. The energy and chemical inputs required for wastewater treatment, as reported by the US EPA, were utilized to model a life cycle inventory of sewer treatment [33].

For CT-SS and UD-SS, the energy consumption required for septic tank cleaning and transportation of residuals from the septic tank was estimated at 5 MJ·(year·household)^−1^ and 68 MJ·(year·household)^−1^, respectively. These energy estimates for a septic tank were based on the assumption that a 2500 gallon vacuum truck was utilized to clean a septic tank every 3 years [2]. Based on consultation with multiple toilet manufacturers (such as Sun-mar and Phoenix), the average electrical load for operating a fan for the composting toilets was 5 W·d^−1^. The median energy use for transporting compost from CT-SS and urine from UD-SS was estimated to be 440 MJ·household^−1^·year^−1^ and 1280 MJ·household^−1^·year^−1^, respectively. These estimates were based on production of 0.5–2.5 L of urine per adult per day [34] and flush water volumes of 0.2–0.6 L·flush^−1^ [24,35]. The author assumed a 3 m^3^ urine storage tank, based on 20 L·d^−1^, 70% of urine collection rate, and 3 months of storage time prior to transport. Further, it was assumed that urine and compost were transported over 200 km from the household to farms, which are located in less nutrient sensitive watershed and use urine and compost as fertilizers. In addition, the nutrient contents of compost and urine are estimated based on previous lab and field experiments [36,37], as listed in Table 1.

The energy use and environmental emissions for manufacturing pipes and construction materials were based on Xue et al. [2]. The energy used to dig the trenches for constructing water and wastewater infrastructure was estimated according to the evacuation volume and diesel requirement by a John Deere 135G excavator (John Deere, Moline, IL, USA). The associated air pollutants were estimated based on diesel consumption and the NONROAD model [38].

The background life cycle inventory was primarily compiled by utilizing the ecoinvent v3.5 database (the ecoinvent centre, Zurich, Switzerland) [39], which is one of most comprehensive life cycle inventory databases. The life cycle inventory was compiled and computed through OpenLCA version 1.8 (GreenDelta, Berlin, Germany), in agreement with the ISO standard for LCA [40]. OpenLCA was chosen due to its transparency and transferability.

#### 2.2.3. Life Cycle Impact Assessment

Characterization factors for life cycle health impacts were obtained from the USEtox model. USEtox is a model officially endorsed by United Nations Environment Program/Society of Environmental Toxicology and Chemistry (UNEP/SETAC) for comparative assessment of human health impacts of chemicals in conjunction with LCA modeling. USEtox’s characterization factors are based on a combination of multimedia environmental fate, multi-pathway exposure assessment, and toxicity assessment models, and often expressed in cases/kg of released chemicals. The life cycle cancer impact of a water service system was equal to the sum of products between amounts of released chemicals and their cancer characterization factors. Similarly, the life cycle noncancer impact of a water service system was equal to the sum of products between amounts of released chemicals and their noncancer characterization factors.

#### 2.2.4. Life Cycle Interpretation

Monte Carlo analysis (MCA) was used to quantify the variability and uncertainty of input parameters contributing to life cycle health impacts. Input parameters included chemical and energy use in centralized water treatment, energy use in water distribution, energy use in wastewater collection, chemical and energy use in centralized wastewater treatment and decentralized septic tanks, and energy use in transporting compost and urine (Table 1). When sufficient datasets were available, best-fit probability distributions were simulated for the input parameters. Otherwise, triangle distributions with max, most likely, and min values were assigned based on the available datasets. Anderson Darling sampling methods were used with over 10,000 iterations in a model constructed for variability assessment. A sensitivity analysis was performed, by perturbing each variable while holding other variables constant at their reference case values, to aid in the understanding of relative influences of input parameters on life cycle impacts, and to provide a basis for prioritizing future data collection efforts and designing mitigation efforts.

### 2.3. QMRA

QMRA approach was used to estimate the human health risks associated with the exposure to fecal pathogens mainly induced by the insufficient operation of the wastewater treatment under BAU, CT-SS, and UD-SS. QMRA typically consists of four steps, including hazard identification, exposure assessment, dose-response assessment, and risk characterization. Hazard identification determines the pathogens and human health interests. Exposure assessment quantifies pathogen doses during exposure events. Dose-response assessment determines the relationship between the exposure dose and the likelihood of the health outcomes. Risk characterization calculates the likelihood of the health outcomes. All four steps are described in detail below.

#### 2.3.1. Hazard Identification

Four prevalent pathogens related with wastewater effluent or poorly treated sewage were selected in this QMRA, including Campylobacter jejuni, Cryptosporidium spp., E. coli O157:H7, and norovirus. These pathogens are responsible for diarrhea and were also assessed in other QMRA studies regarding sanitation services [45].

#### 2.3.2. Exposure and Dose Response Assessment

While a range of exposure scenarios can occur, this study focused on exposure scenarios, which may cause substantial differences among the three studied systems. This study excluded the exposure scenarios that lead to the same health risks among the three systems. For example, pathogen exposure during the use phase of the examined toilet systems has been excluded, because this exposure scenario poses the same risks across the three systems. Moreover, the contact with the fecal matter during the defecation process has also been excluded for all examined systems. Furthermore, the cross-contamination of the human urine has been omitted, as storage over 3 months can render urine sanitized and in turn safe for agricultural use [46,47]. Additionally, the risk associated with using compost as soil amendment was excluded. Most prior studies indicated a 100% removal of pathogens for compost as soil amendment, with few exceptions showing Helminth eggs in the manure compost [12]. Due to a lack of international standards on quantitatively assessing health risks of Helminth eggs, the health risk of using compost as soil amendment was not included [12]. For comparison purposes, this study focused on the two exposure pathways of accidental ingestion of recreational water contaminated with discharge from a wastewater treatment plant in BAU or from septic tanks in CT-SS and UD-SS, and ingestion of household potable water contaminated due to cross-connection to sewage in BAU.

The dose of each pathogen by accidental ingestion of recreational water contaminated by BAU’s discharge or CT-SS and UD-SS’s septic effluent was calculated as Equation (1).

D_p, j_ = V_i_/1000 × Dil_j_ × 10^(C_j,p_ − R_j,p_)^ (1)

where D_p, j_ is the dose of pathogen p under system j. p is Campylobacter jejuni, Cryptosporidium spp., E. coli O157:H7, and norovirus. j is BAU, CT-SS and UD-SS. V_i_ is the volume of water ingested (mL). Dil_j_ is the dilution factor for system j’s discharge in recreational water. C_j,p_ is the influent concentration for pathogen p for system j (log(genome or oocyst/L)). For example, C_BAU,p_ is the influent concentration for pathogen p under BAU. C_CT-SS,p_ is the influent concentration for pathogen p under CT-SS. C_UD-SS,p_ is the influent concentration for pathogen p under UD-SS. R_j,p_ is log removal/inaction for pathogen p for system j. For example, R_BAU,p_ is log removal/inactivation for pathogen p under BAU. R_CT-SS,p_ is log removal/inactivation for pathogen p by soil transportation under CT-SS. R_UD-SS,p_ is log removal/inactivation for pathogen p by soil transportation under UD-SS.

For BAU, the pathogen concentrations in wastewater influent (C_BAU,p_), the pathogen removal efficiencies of wastewater treatment plant (R_BAU,p_) and volume of water ingested (V_i_) are from previous U.S. EPA studies [45,48]. Additionally, the dilution factor for BAU was based on the existing literature [11]. Consistent with the existing literature, the pathogen density in septic leakage from UD-SS was considered to be equivalent to raw wastewater due to sufficient leakage from multiple septic tanks [11]. Two exceptions are the septic leakage dilution Dil_UD-SS_ and norovirus removal R_UD-SS,n_. Since the dilution factor varies based on the volume of leakage and the characteristics of the recreational waterbody, it is characterized by a uniform distribution with a lower limit of 1/100 and an upper limit of 1/10. The removal of the norovirus present in the leakage by soil transport also varies depending on the soil conditions and travel distance [49]. Consistent with the existing studies [49,50], the norovirus removal through the sandy soil in Falmouth is characterized with a log-uniform distribution. The ingested volume, dilution factor, and septic removal rate for CT-SS were identical to UD-SS, except for the norovirus concentration in septic effluent (C_CT-SS,n_). The norovirus density in septic tank effluent was characterized with a uniform distribution, based on previous studies [4,11].

The dose of ingested household potable water contaminated due to cross-connection to sewage in BAU for each pathogen was calculated for each day over the duration of the event:

D_p_ = V_p_ × Dil_cc_ × (10^C_BAU,p_^) × 10^(−k_p_ × C_d,c_ × t)^ (2)

where V_p_ is the volume of potable water ingested per day (L/day). Dil_cc_ is the cross-connection dilution factor. k_p_ is Chick–Watson inactivation constant for pathogen p (mg min/L)^−1^. C_d,c_ is the concentration of chlorine in the distribution system (mg/L). t is the contact time (min).

The characterization of the pathogen densities in the raw wastewater (C_BAU,p_) and the volume of water ingested (V_p_) are as previously reported in U.S. EPA studies [45,51]. The inactivation constant (k_p_) was derived by Teunis, et al. [52] in a QMRA of negative pressure events. A conservative dilution factor (Dil_cc_) of 0.001 was used to reflect the wastewater intrusion during pressure events. The contact time (t) and concentration of chlorine (C_d,c_) in the system are based on the previous studies [11].

The estimated doses were used as inputs in the relevant dose-response relationships, shown in Table 2, to provide predicted probabilities of infection. The probabilities of infection multiplied by the probabilities of illness given infection were used to estimate the illness risk for each dose. The distribution of annual illness risk (P_illa_) was estimated assuming one event per year over the duration of the event as:

P_illa_ = 1 − [(1 − P_ill1_) × (1 − P_ill2_) × … × (1 − P_illn_)] (3)

#### 2.3.3. Risk Characterization

Risk characterization entails the integration of the information provided from the aforementioned hazard characterization, dose-exposure, and dose-response steps, to quantify the effects on human health in disability-adjusted life years (DALY). The equation employed for the estimation of the total burden of disease in DALY/year is given by Equation (4) [12,45].

DALY_a_ = P_illa_ × P_exp_ × DALY_case_ (4)

where DALY_a_ is annual total burden of diseases. P_illa_ is the probability of annual illness risk. P_exp_ is the exposed population. DALY_case_ is DALY per case of illness.

The annual DALY (DALY_a_) for each exposure pathway is the product of the estimated probability of annual illness (P_illa_), the exposed population (P_exp_), and the DALY per case of illness (DALY_case_) [11,12]. On the basis of a national survey of 75,000 households, the participation rate of persons 16 years of age or older for swimming was 25% per year. The percent of the population affected by a cross-connection event is uncertain and was set at 10% as a conservative estimate. The DALY per case of illness used in this study is 4.6×10^−3^ for C. jejuni [58], 1.7×10^−3^ for Cryptosporidium [59], 5.5×10^−2^ for E. coli O157:H7 [60], 1.6×10^−3^ for norovirus illness attributed to swimming and 9.5×10^−4^ for norovirus illness attributed to cross contamination [61].

### 2.4. Integration of LCA and QMRA

In order to aggregate the results generated from LCA in Section 2.2 and QMRA in Section 2.3, a unit of DALY/year was used. The relationship between cases of illness and DALY was used to convert the life cycle cancer and noncancer impacts. one case of illness is equivalent to 11.5 DALY for cancer impact, while 1 case of illness is equivalent to 2.7 DALY for non-cancer impact [62]. The annual microbial health risks were determined in 2.3 with the unit of DALY/year. The total health risk from both LCA and QMRA was the sum of health impacts from both LCA and QMRA in the unit of DALY/year.

## 3. Results

### 3.1. Magnitudes of Life Cycle Health Impacts

The median life cycle cancer impact ranged from 0.0018 to 0.0066 DALY/year for three assessed water systems. BAU presented the highest median life cycle cancer impact, amounting to approximately 0.0066 DALY/year. In contrast, UD-SS showed the lowest median life cycle cancer impact of 0.0018 DALY/year. Meanwhile, the median life cycle noncancer impacts spanned from 0.017 to 0.047 DALY/year. Consistently, BAU and UD-SS showed the highest and lowest median life cycle noncancer impacts, respectively. The discrepancy in life cycle health impacts of the three systems was mainly due to differences in their energy use. BAU was the most energy intensive system among the three systems. CT-SS and UD-SS’s life cycle energy use was only 20% and 40% of BAU’s life cycle energy use, respectively. Energy production and consumption generate a range of toxic releases into the air, water, and soil compartments (such as NOx, SOx, particulate matter and aqueous and solid disposal). In general, the recovery-based service options UD-SS and CT-SS used much less energy than BAU, and therefore showed lower life cycle health impacts than BAU.

### 3.2. Relative Contributions of Life Cycle Health Impacts and Microbial Health Risks

**Figure 2:**
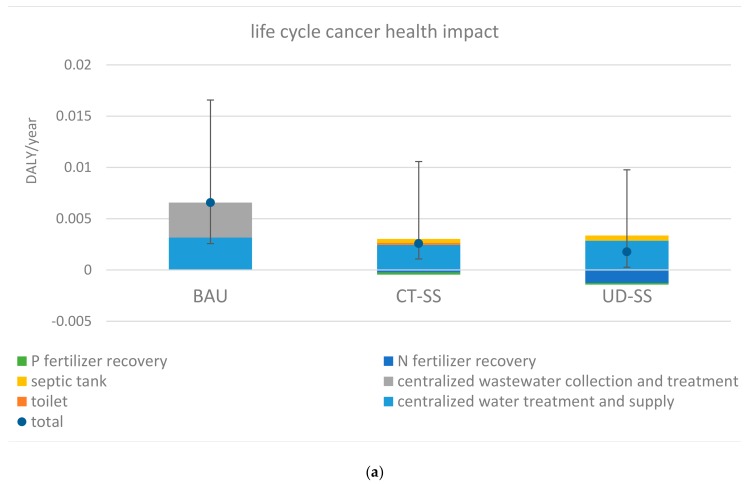

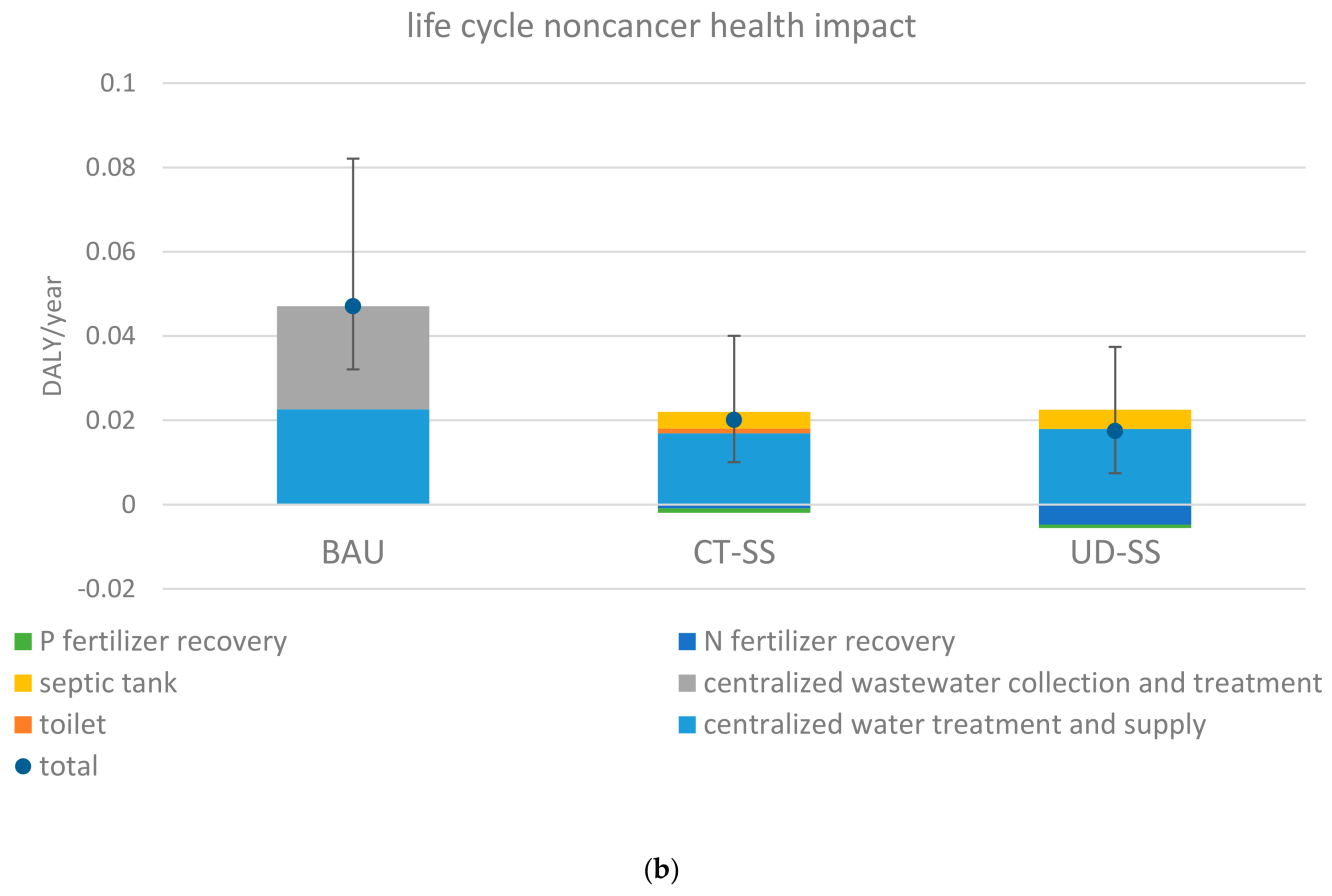

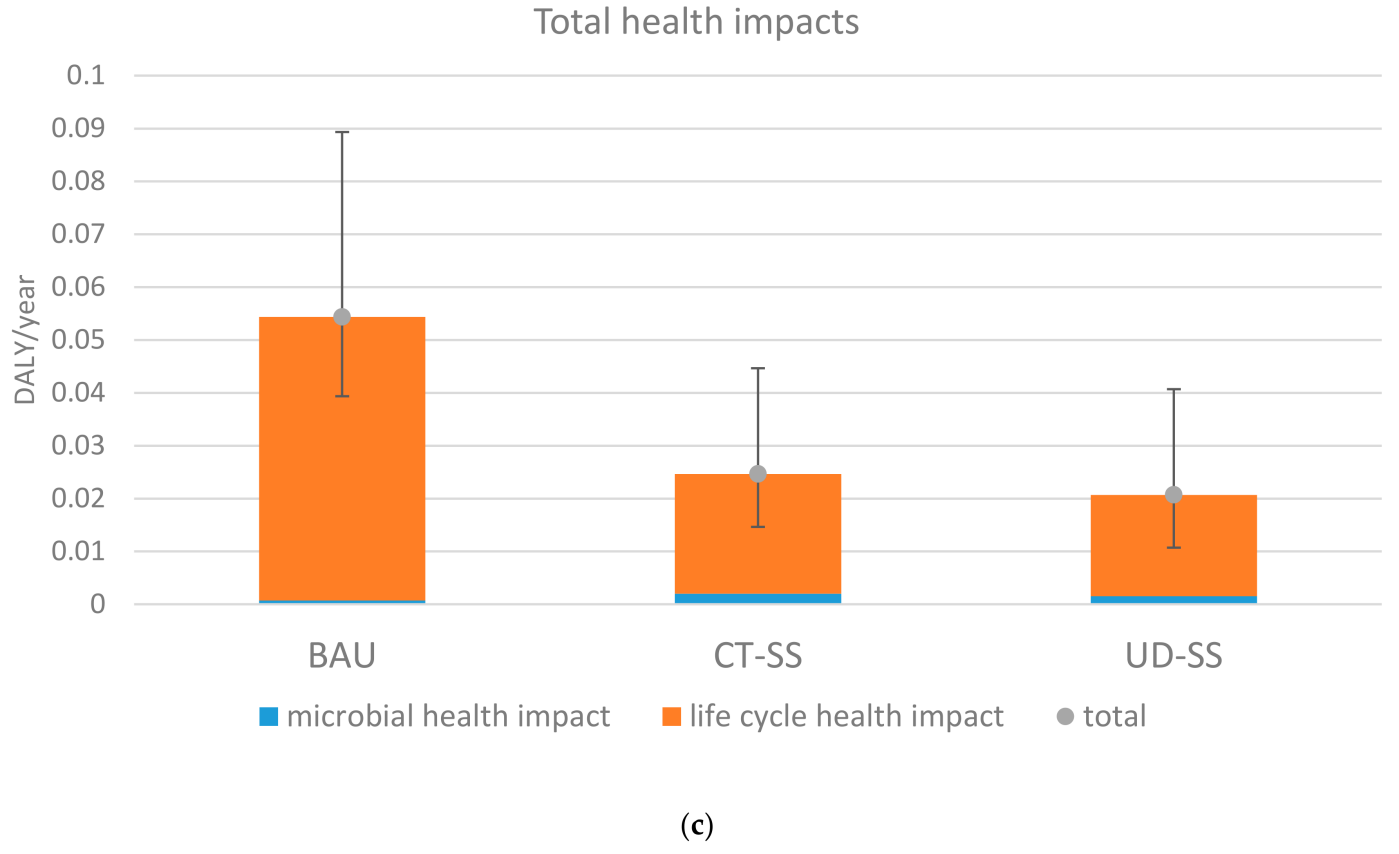
**(a)** Life cycle cancer health impact of BAU, CT-SS, and UD-SS, DALY/year. The range of variability bar presents the values at 5th and 95th percentiles. (**b**) Life cycle noncancer health impact, DALY/year. The range of variability bar presents the values at 5th and 95th percentiles. (**c**) Life cycle and microbial health impacts, DALY/year. The range of variability bar presents the values at 5th and 95th percentiles.

The contributions of treatment stages to life cycle noncancer impact varied among BAU, CT-SS, and UD-SS (Figure 2a,b). For BAU, municipal wastewater collection and treatment stages accounted for 52% of the life cycle noncancer impact, slightly exceeding the contribution of the municipal water treatment and delivery stage. For CT-SS and UD-SS, municipal water treatment and delivery was the dominating contributor, resulting in over 85% of their total life cycle noncancer impact. Following the municipal water supply stage, the septic treatment stage contributed to approximately 20% of the total life cycle noncancer impact for CT-SS and UD-SS. Nutrient production from compost reduced approximately 10% of life cycle noncancer impact for CT-SS. Nutrient production from urine mitigated approximately 30% of life cycle noncancer impact. The contributions of treatment stages to life cycle cancer impact were similar in magnitude to their contributions to life cycle noncancer impact. Similarly, the contribution of the municipal wastewater collection and treatment stage to the life cycle cancer impact (54%) was slightly higher than the contribution of the municipal water treatment and delivery stage. The municipal water treatment and delivery stage was the leading contributor to the life cycle cancer impacts of CT-SS and UD-SS. Nutrient production from compost and urine offset were 11% and 35% of the life cycle cancer impacts for CT-SS and UD-SS, respectively. Furthermore, comparing the relative shares of the construction and operational phases for life cycle cancer and noncancer impacts, the operational phase ranked as the dominating contributor (more than 98%) for all three systems. Additionally, the life cycle health impact dominated the total health impact for all three systems (Figure 2c). The relative contributions of life cycle health to total health impact exceeded 95% for all three assessed systems. Microbial risks contributed to less than a mere 5% of total health impacts.

### 3.3. Variability and Uncertainty Analyses

The assessed water systems exhibited considerable variability and uncertainty in life cycle health impacts (Figure 2a–c). The life cycle cancer impact of BAU ranged from 0.003 to 0.017 DALY/year. The life cycle noncancer impact of BAU varied from 0.03 to 0.08 DALY/year. BAU’s total health impact, including both life cycle and microbial health impacts, spanned from 0.04 to 0.09 DALY/year. These large spans were due to both natural variability and data uncertainty. First, large variations exist in the life cycle health impact intensity of different energy sources. The ecoinvent database indicated that the life cycle noncancer impacts of coal could be 100 times higher than that of solar, in terms of cases/MJ energy. Second, the electricity use of water and wastewater treatment varied across geographical areas, treatment processes, and operational conditions. Xue et al. estimated that the electricity use of centralized wastewater collection and treatment processes ranged from 0.17 to 1.4 kwh/m^3^ treated wastewater [1]. Furthermore, uncertainty exists in toxic release inventory. Although the life cycle chemical release inventory has rapidly grown, large uncertainty exists in the chemical release profiles. For example, the pesticide release from oil crop for bioenergy production differed by a factor of 20, due to utilizing different input datasets and models [63]. Additionally, uncertainty is embedded in the microbial risk assessment. For example, the microbial concentration and affected population are uncertain for the ingestion of drinking water contaminated by a long duration and high dilution cross-connection to sewage [11].

## 4. Discussion

### 4.1. Sensitivity Assessment

**Figure 3:**
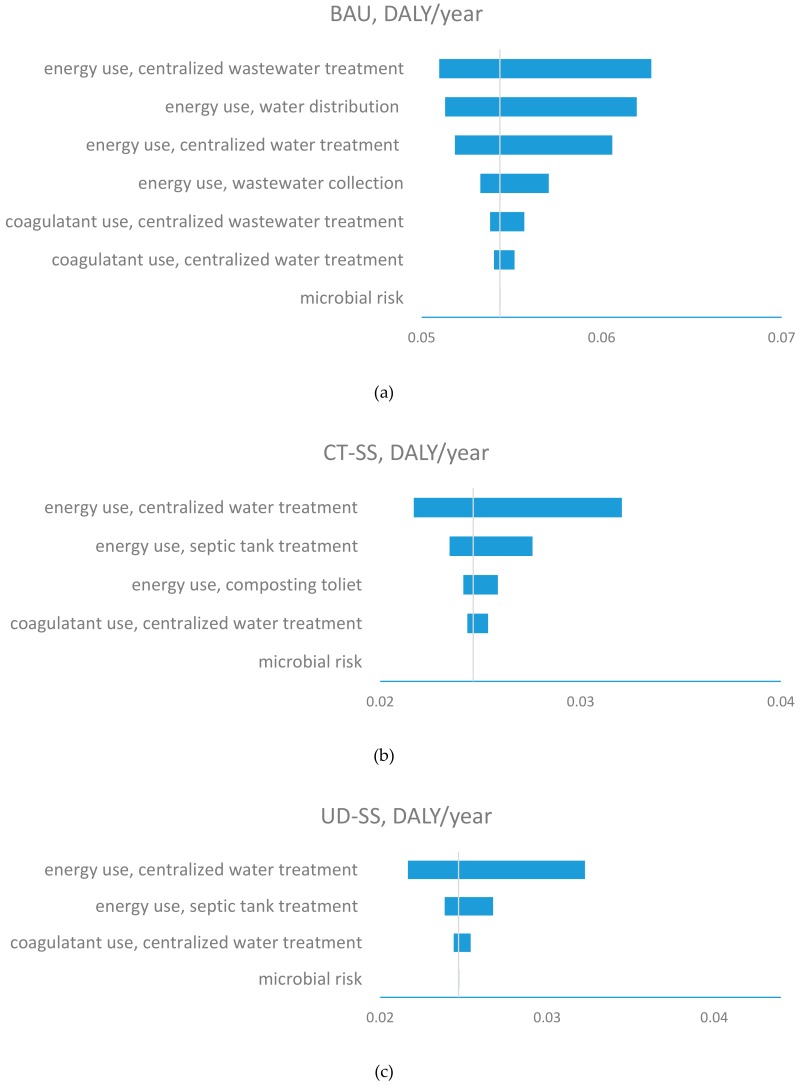
Sensitivity of health impacts for BAU, CT-SS, and UD-SS. Vertical lines represent the base values for each system. The bars demonstrate the variations in total health impacts corresponding to the ranges of inputs parameter such as energy/coagulant use and microbial risk. (**a**) BAU; (**b**) CT-SS; (**c**) UD-SS.

The sensitivity analyses indicated that BAU’s total health risk, in terms of DALY/year, were mainly driven by energy use during wastewater treatment, water distribution, and water treatment (Figure 3a). Energy use of the wastewater treatment plant was the most influential factor for total health risk of BAU. BAU’s total health risk varied from 0.051 to 0.063 DALY/year, corresponding, respectively, to 5^th^ and 9^th^ percentiles of energy use of the wastewater treatment plant. The second most influential parameter for BAU was energy use for water distribution. The total health risk of BAU ranged from 0.051 to 0.063 DALY/year, corresponding to 5^th^ and 9^th^ percentiles of energy use during energy use of water distribution. As follows, energy use for water treatment ranked as the third greatest contributor to the total health risk of BAU. Additionally, varying energy use for wastewater collection, chemical use for water and wastewater treatment, and microbial health impact resulted in a change of less than 5% in total health risk of BAU.

Differing from BAU, energy use of water treatment ranked as the most influential factor for the total health risk of CT-SS. The total health risk of CT-SS varied from 0.021 to 0.032 DALY/year, corresponding to 5^th^ and 9^th^ percentiles of energy use during water treatment. Energy use for septic tank ranked as the second most influential factor for the total health risk of CT-SS. Additionally, the energy use for transporting compost and the coagulant use for water treatment had insignificant influences on the total health risk. Similarly, energy use of water treatment and septic tanks were the top two influential factors for the total health risk of UD-SS. The total health risk of UD-SS ranged from 0.017 to 0.028 DALY/year. Varying the coagulant use for water treatment and the microbial risk resulted in less than 5% variation in total health risk of UD-SS.

### 4.2. Implications for Infrastructure Management and Policy Development

This study emphasized the importance of including both life cycle and microbial health impacts for comparing health risks of different sanitation systems. First, coupling LCA and QMRA provides a more holistic understanding of health impacts of water and sanitation systems. The LCA and QMRA approaches had different foci, but are complementary to each other. Life cycle health impacts were mainly associated with chemical releases during upstream material/energy production, which usually occur far away from the wastewater treatment facility and outside of the service community. Microbial health impacts were mainly resulted from local exposure to pathogens within the service community. Coupling LCA and QMRA approaches provided total health impacts from exposure to both chemical and microbial pathogens, and from water and sanitation processes and their supply chain activities, such as material/energy production. Second, it is important to note that life cycle health impacts dominated the total health impacts. Without considering life cycle health impacts, the total health impacts would be significantly underestimated. This finding highlighted that life cycle health impacts of supply chain processes (such as energy and material use for constructing and operating water and wastewater infrastructure) must be considered when designing civil infrastructure and health management policies in order to avoid causing negative health consequences from supply chain activities.

The stage contribution analyses suggested targeted mitigation strategies for effectively reducing negative health impacts of three different systems. First, considering the dominating contribution of life cycle health impact to total health impact, reducing energy use and switching to cleaner energy sources (such as solar and wind energies) would be capable of significantly reducing life cycle health impacts and total health impacts of three different systems. Second, different water treatment stages should be targeted for effectively mitigating negative health impacts of the three water systems. The wastewater treatment stage was the largest contributor to the total health impacts of BAU and offers promising opportunities for mitigating negative health impacts. For example, reducing energy use of wastewater treatment processes in BAU would be effective in reducing life cycle health impacts of BAU. In contrast, the water treatment stage ranked as the top contributor to the total health impacts of CT-SS and UD-SS, suggesting reducing energy use of water treatment processes would be effective in reducing health impacts of CT-SS and UD-SS. Additionally, preventing cross contamination and recreational risks would be effective in mitigating local microbial risks for BAU, CT-SS, and UD-SS. Replacing/maintaining aging water delivery infrastructure and implementing utility-wide monitoring and prevention programs will aid in mitigating microbial risks from cross contamination for BAU. Community campaigns and education programs would be valuable to mitigate the recreational risks for local swimmers.

Last, while public health risk is an important criterion for selecting community water and wastewater service options, environmental and cost considerations play critical roles in decision making as well. The syntheses of the existing literature and this study indicated that the decentralized design options such as CT-SS and UD-SS presented lower energy use, cost, greenhouse gas emissions, nutrient releases, and health risks than the traditional centralized BAU [2,3,4,6]. However, tradeoffs exist between the decentralized design options, highlighting the inherited difficulty in simultaneously reducing cost and environmental and health damages. CT-SS showed slightly lower energy use, greenhouse gas emissions, nutrient releases, and water use than UD-SS. In contrast, UD-SS had slightly lower cost and total health risks than CT-SS. Overall, comprehensive evaluation of water and wastewater treatment systems from economic, environmental, social, and technological aspects is required to support the design and use of sustainable water and sanitation services.

## 5. Conclusions

Understanding the health impacts of water and wastewater systems is critical to aiding in the design and implementation of sustainable water and sanitation services. Based on the integrated LCA and QMRA approaches, this study found that CT-SS and UD-SS outperformed BAU from both life cycle health and microbial risk perspectives. Life cycle health impacts dominated the total health impacts for all three systems. The wastewater treatment stage ranked as the top contributor to the life cycle health impacts of BAU. The water delivery stage was shown as the most significant contributor to the life cycle health impacts of CT-SS and UD-SS. The variability and sensitivity assessment suggested that reducing energy use of the top contributing stages was effective in reducing life cycle health impacts. Overall, comprehensive assessments of water and wastewater systems across multiple sustainability indicators should be conducted in order to support the maximization of environmental benefits and the minimization of health damages of water and wastewater systems.

## Figures and Tables

**Table 1:** Data sources, distributions, and ranges for key input parameters.

Input Parameter	Input Statistic	Range for the Sensitivity Analysis		References
	Distribution ^+^	Low (5th Percentile)	High (95th Percentile)	
Coagulant input of water treatment, kg·m^−3^ treated water	Triangular (0.016, 0.019, 0.028)	0.017	0.026	[1,41,42]
Energy use of water treatment, MJ·m^−3^ water	Normal (1.27, 0.43)	0.56	1.98	[1,41,42]
Energy use of water distribution, MJ·m^−3^ water	Normal (0.95, 0.41)	0.28	1.62	[1,41,42]
Energy use of wastewater collection for BAU, MJ·m^−3^ wastewater	Normal (0.47, 0.24)	0.08	0.86	[1,41,42]
Coagulant input of centralized wastewater treatment plant for BAU, kg·m^−3^ wastewater	Triangular (0.008, 0.010, 0.012)	0.09	0.011	[1,33]
Energy use of centralized wastewater treatment plant for BAU, MJ·m^−3^ wastewater	Normal (2.34, 0.6)	1.35	3.33	[1,41,42]
Energy use of septic treatment for CT-SS and UD-SS, MJ·m^−3^ wastewater	Normal (0.18,0.03)	0.13	0.23	[34,43,44]
Energy use of transporting compost for CT-SS, MJ·(household·year)^−1^	Normal (440, 30)	391	489	[36,37]
Energy use of transporting urine for UD-SS, MJ·(household·year)^−1^	Normal (1280, 100)	1116	1445	[36,37]

^+^ Parameters are in parentheses, in this order: for the normal distribution (mean and standard deviation); for the triangular distribution (minimum, peak, and maximum).

**Table 2:** Reference Hazards and Dose-Response Models.

Reference Hazard	Dose-Response Model	Parameters	ID50	Pill | Inf
Campylobacter jejuni	Beta-Poisson [53,54]	0.145; 7.59	800 cfu	0.33
Cryptosporidium spp.	Exponential [55]	0.09	8 oocysts	0.71
E. coli O157:H7	Beta-Poisson [56]	0.4, 45.9	207 cfu	0.28
Norovirus	Hypergeometric [57]	0.04, 0.055	26 genome copies	0.7
